# Out-of-pocket expenditure and catastrophic health expenditure for hospitalization due to injuries in public sector hospitals in North India

**DOI:** 10.1371/journal.pone.0224721

**Published:** 2019-11-07

**Authors:** Shankar Prinja, Jagnoor Jagnoor, Deepshikha Sharma, Sameer Aggarwal, Swati Katoch, P. V. M. Lakshmi, Rebecca Ivers

**Affiliations:** 1 Post Graduate Institute of Medical Education and Research, Chandigarh, India; 2 The George Institute for Global Health, New Delhi, India; 3 The George Institute for Global Health, University of Sydney, Sydney, Australia; University of South Australia, AUSTRALIA

## Abstract

**Background:**

Injuries are a major public health problem, resulting in high health care demand and economic burden. They result in loss of disability adjusted life years (DALYs) and high out-of-pocket expenditure. However, there is little evidence on the economic burden of injuries in India. We undertook this study to report out-of-pocket expenditure and the prevalence of catastrophic health expenditure for injuries related hospitalizations in public sector hospitals in North India. Further, we also evaluate the determinants of catastrophic health expenditure.

**Methods and analysis:**

A prospective observational study was conducted. Participants were recruited from three hospitals for all injury cases. Data were collected via face-to-face baseline interviews and follow-up interviews over the phone at 1, 2, 4 and 12 months post-injury. Prevalence of catastrophic health expenditure (more than 30% of consumption expenditure) and impoverishment (International dollar 1.90) were estimated.

**Results:**

Road traffic injuries (57%) were the leading cause of injury. Direct out-of-pocket expenditure for hospitalizations was INR 16,768 (USD 263) while indirect productivity loss was INR 8,164 (USD 128). The prevalence of catastrophic expenditure was 22.2% with 12.2% slipping below poverty line. Prevalence of catastrophic health expenditure and impoverishment was higher and significantly associated with poorest quintile, tertiary care hospital and increased duration of hospitalization (p< 0.001).

**Conclusion:**

The economic impact of injuries is notably high both in terms of out-of-pocket expenditure and productivity loss. A high proportion of households experienced catastrophic expenditure and impoverishment following an injury, highlighting need for programs to prevent injuries.

## Introduction

Globally, every year, 973 million people sustain some form of unintentional injury while 4.8 million people die as a result of injury [1]. This accounts for 9% of the world’s deaths, nearly 1.7 times the number of fatalities that result from HIV/AIDS, tuberculosis and malaria combined [2]. Unintentional injuries including road traffic injuries (RTI), falls, burns and drowning account for about three quarters of injury related deaths [3]. RTIs in particular are the leading cause of injury related deaths, disabilities, hospitalizations and socioeconomic losses with nearly 1.36 million deaths worldwide in 2015 [4]. Injuries in general have been ranked as the leading cause of disability-adjusted life years (DALYs) lost among economically productive group of 15- to 29-year-olds [5].

Injuries have a significant economic burden, when viewed both from the microeconomic or macroeconomic lens, as they impose heavy costs on the individual and the society. The cost of injuries in India is estimated to be between 2–3% of the gross domestic product (GDP) [6]. At the household level, these result in very high out-of-pocket payments, pushing the families of the victims to face financial catastrophe. National sample survey (NSS) reports average out-of-pocket (OOP) expenditure per injury-related hospitalization case in India to be INR 23,491(USD 368.9) [7]. Other cross-sectional studies conducted in various parts of the country estimated average OOP expenditure for RTI to range from US$ 100 to US$780 [8]-[11]. Further, injury-related OOP expenditures are often catastrophic in nature. Prevalence of catastrophic health expenditure (CHE) among RTI and non RTI patients has been reported to be 27.0% and 34.4% respectively [11]. Another recent study found the odds of facing CHE for injuries to be 4.03 (3.16–5.15 p <0.001) times higher than communicable diseases [12].

Apart from the direct OOP expenditure, victims also incur indirect costs in terms of lost productivity (work loss) on account of both the victim as well as the caregivers. Despite this, the majority of the literature does not include lost productivity and workplace losses, and so lack a valid and complete estimate of the economic burden of injuries [13]. A systematic review was conducted by Wesson et al, on the extent and quality of economic evidence related to injuries in lower and middle income countries. It reported that, majority (49%) of the studies were hospital based and one in every two studies on injuries described RTIs only. Further, only 32% of the studies included indirect costs besides direct expenditure (medical and non-medical) [14]. Reddy et al, examined both direct and indirect costs related to traffic crashes, however, this study had a very small sample size (95 crashes) without inclusion of follow-up costs [8]. A large scale household level survey undertaken in Bangladesh and Bangalore to assess the incidence and impact of RTIs, explored the aspect of productivity losses. While, the study reports on the extent of working days lost as a result of RTI (which ranged from 89 days to 124 days in Bangladesh and 133 days to 180 days in Bangalore), it did not quantify the magnitude of economic losses as a result of lost productivity [10]. Majority of previous research has reported on the economic burden associated with road traffic crashes only [8]-[11], [15], [16]. Moreover, a complete assessment of financial impact is also lacking as studies include expenditure incurred only at the time of hospitalization and the patients are not followed up beyond discharge. The after discharge period might involve the major portion of the expenditure in many cases. Results from our earlier study indicate that mean OOP expenditure over 12 months, following discharge, were 2.5 times the OOP amount incurred during hospitalization [11].

We undertook this study to assess the extent of direct OOP expenses and indirect costs due to injury-related hospitalization including costs up to 12 months post discharge. We also assess the prevalence of catastrophic health expenditure and change in poverty head count i.e. impoverishment as a result of OOP expenditure.

## Methodology

### 2.1. Study settings

The study protocol has been previously published [13]. Patients were recruited from three health care institutions: the Postgraduate Institute of Medical Education and Research (PGIMER), a tertiary care institution located in Chandigarh; Government Multi-Specialty Hospital (GMSH), a secondary care institution also located in Chandigarh; and Government Hospital, a secondary care institution located in Panchkula district of Haryana. The choice of these three hospitals was purposive, as these catered to large volumes of trauma patients. Patients were recruited for the present study from April to August 2015 from the emergency ward/ trauma care centre of the three hospitals.

The *Postgraduate Institute of Medical Education and Research (PGIMER)* is a large 1850 bed super specialty hospital. The Advanced Trauma Centre (ATC) of the institute is a specialized and fully equipped centre catering to the needs of trauma victims. Besides the injury patients who come to ATC directly, this hospital serves as a referral centre for hospitals in Chandigarh and neighboring states such as Punjab, Haryana, Himachal Pradesh, Uttar Pradesh etc. ATC is equipped with an out-patient department, an in-patient department, operation theatre and diagnostics services including all necessary radiological and pathological tests. A total of 5,392 trauma patients received care at the trauma centre during 2014–15.

*Government Multi-Specialty Hospital* is a 500 bed secondary care hospital providing care to the residents of Chandigarh and the surrounding states. A total of 1,574 trauma cases received care during 2014–15.

*District level hospital* is a 300 bed secondary care hospital located in Panchkula district of Haryana. Trauma patients are treated along with other emergency patients at the emergency centre of the hospital. Severe trauma injury patients in this hospital are referred to other tertiary care hospitals in the region. A total of 11,511 trauma patients received care at district hospital during 2014–15.

### 2.2. Study design

A prospective cohort study approach was used to recruit 2955 participants of all ages injured due to various causes like road crashes, falls, burns, mechanical injuries, animal bites, poisoning, drowning etc. requiring overnight hospitalization at the three participating hospitals. Overnight hospitalization was defined as a stay of over 12 hours in the hospital, with the patient being admitted and discharged on different dates.

Sample size was estimated assuming a prevalence of catastrophic health expenditure rate to be 22% from a previous study [9], with a precision of 1% and a confidence interval of 95%. Based on these assumptions, a sample size of 2955 was considered appropriate. The sample distribution in PGIMER, Government Multi-Specialty Hospital, and the district level hospital was 2327, 553 and 75 respectively.

Patients were interviewed using a pretested structured questionnaire. Face to face interviews were conducted at the time of hospitalization while follow up interviews were conducted over the phone at 1, 2, 4 and 12 months after discharge [17].

### 2.3. Data collection

During baseline interviews, detailed information was collected from patients or primary caregivers on general socio-demographic characteristics of the patient such as age, gender, education, income, household consumption expenditure. Household consumption expenditure was defined as the total monetary values of all the items (i.e., goods and services) consumed by the household on domestic account during the reference period, inclusive of the imputed values of consumption of goods and service which are not purchased but procured otherwise for consumption [18]. However, this data could not be collected in 28% of the cases. The respondents were interviewed for type and mechanism of injury, treatment details, length of hospital stay etc. Finally, data were also collected on a daily basis during hospitalization until discharge regarding OOP expenditure during the last 24 hours. This included OOP expenditure for user fees, medicines, diagnostics, procedure charges, transportation, informal charges and others. User fees included any fees that were charged by the hospital for doctor consultation, supply and medical material costs, bed charges etc. Procedure charges refer to fee charged by the hospital for any major or minor surgical procedure undertaken. Any form of expenditure which was incurred but the patient was unable to classify it into any of the above stated categories was analyzed under any other expenditure. OOP health expenditure was also elicited during follow up interviews. Data on indirect costs were elicited by interviewing both patients and their caregivers. Indirect costs refer to the value of time lost because the patient and the caregivers are unable to carry out normal productive activities because of injury. Patients and all caregivers (one or multiple) accompanying the patients during the period of hospitalization were interviewed to estimate indirect costs.

#### Post-hospitalization data collection

**Fig 1:**
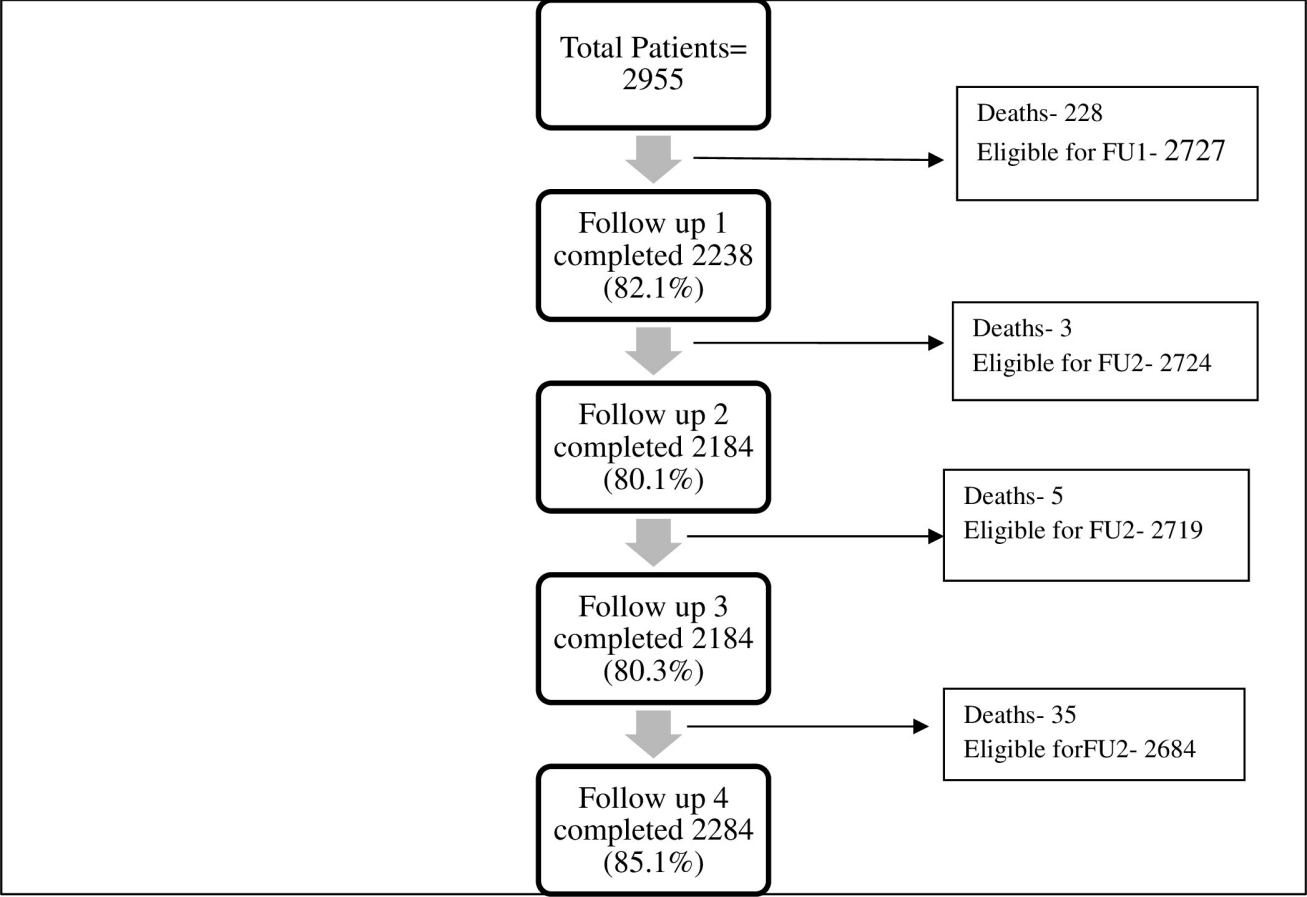
Flowchart showing follow-up (FU) rates.

During the follow-up interviews over the phone at 1, 2, 4 and 12 months, OOP expenditure incurred on medicines, diagnostics, transportation and user fees (in case of follow up visits) since the period of discharge/ last interview was recorded. The number of patients who were contacted and interviewed during follow-up period is shown in Fig 1. More than 85% of all eligible patients were interviewed at 12 months following discharge. (S1 Dataset)

### 2.4. Data analysis

Data were analyzed using SPSS version 21. Descriptive statistics were used to analyze socio-demographic profile of the injury patients. Further chi-squared test was applied to check for the relationship between patients’ socio-demographic characteristics and injury type. Average OOP expenditure (mean and median) incurred for the period of hospitalization due to injury and up to 12 months post discharge for various types of injuries was calculated. To assess bivariate associations between the study variables independent t-test and ANOVA were used. The independent study variables were grouped into categories for data analysis. For categorizing age groups programmatic classification was followed. In order to classify education and income standard National Sample Survey Organization (NSSO) classification was used [7]. Hospitalization duration (length of stay) was grouped based on exploratory data analysis with regard to the number of patients in each category. Further, this was also done based on expert consultation as length of stay is an important proxy of severity of injury. The hospital length of stay was grouped into categories (0–3 days, 4–6 days, 7–10 days and >10 days).

The prevalence of CHE and increase in relative poverty head count due to hospitalization as a result of injury was measured. CHE was defined as any OOP expenditure on health care that exceeds 30% of the annual consumption expenditure [11], [13], [19–23]. Further, the threshold was varied from 20% to 40% in the sensitivity analysis. Odds of facing CHE according to socio-demographic and injury related characteristics of the patients were calculated and p-values were computed using chi-squared test. CHE and its determinants were estimated using direct OOP expenditure for the period of hospitalization only.

We also estimated the impoverishment induced as a result of OOP expenditure on hospitalization due to injury using direct OOP expenditure for the period of hospitalization. For this indicator, we calculated the poverty headcounts by computing per capita household consumption expenditure before and after injury. Age and household consumption was adjusted using the OECD equivalence scale [24]. Households with adjusted per capita consumption expenditures below the given poverty threshold of International Dollars (Int$) 1.90 per capita per day were considered below poverty line. Finally, the difference between the pre- and post- injury headcounts provided the impoverishment rate as a result of injury hospitalization.

Multiple logistic regression analysis was performed to identify the factors associated with odds of incurring catastrophic expenditure and impoverishment due to hospitalization. Prevalence of CHE and impoverishment were taken as the dependent variables in two separate logistic regression models. The independent variables included in the model were age of the patient, gender, educational status, health insurance, income quintile, type of hospital, hospitalization duration and type of injury. The probability of CHE was calculated using the following logistic equation [25]

$$Prob[y=1]=\frac{\exp(\beta \prime x)}{1+exp(\beta^{\prime }x)}$$

Where, y = having catastrophic health expenditure; [Yes = 1, Otherwise = 0]

β = a set of parameters to be estimated
*x* = a set of predetermined variables

For calculating indirect costs, human capital approach (HCA) was used [26]. Loss of working days was recorded, that is the days that a person (patient as well as the caregiver) missed or remained absent from his/her work due to hospital stay. For those actively employed in the labor workforce, per day income as reported by the individual was used for calculations. For individuals not part of the workforce, instead of taking per day income as zero, an average minimum daily wage rate for India specific to gender and area of residence (rural/urban) was imputed [27]. This was done in order to avoid any sort of underestimation. Productivity losses for children below the age of 14 years was assumed to be zero, as legal age for working in India is 14 years and above. For any person dying, as a result of injury, indirect costs were calculated at the current wage rate for employed and imputed wage rates for unemployed until the age of retirement (assumed to be at the age of 60).

Additionally, indirect costs were also estimated using friction cost approach (FCA). Contrary to the HCA, FCA is based on the concept of worker replacement and generates more realistic estimates particularly in scenarios where worker replacement from labor reserves is possible. In the FCA, the productivity losses are limited to the working population for a specified time interval referred to as the friction period. The friction period is defined as the time span until which the individual who is absent from work due to illness is replaced by another individual, thus restoring the production levels of the organization [28]. In our calculations we used a friction period of 3 months [29]. Further, it has been reported that the annual labor time is not equivalent to the annual labor productivity. This means that one month of absence from work is not equal to one month of production losses, thus while estimating indirect costs using FCA a correction factor referred to as elasticity is needed [28]. In our analysis, we used an elasticity value of 0.8 which translates into a 10% reduction in labor time results in an 8% decrease in production [29].

### 2.5 Ethical consideration

Written informed consent was obtained from the study participants. In case of inability of the patient to give informed consent, it was sought from the immediate attendant/caregiver accompanying the patient. Ethical approval to undertake the study was obtained from Institute Ethics Committee of the Post Graduate Institute of Medical Education and Research, Chandigarh, India. Administrative approvals to collect data were also obtained from concerned authorities of the respective study hospitals.

## Results

A total of 2955 injury patients were recruited for the study. More than half of the hospitalizations were due to road traffic injuries (RTI) (57%), followed by falls (23%). Most commonly, patients were male (79.3%), aged 16–30 years (38.7%), educated above 10th year of education (44%) and without any form of health insurance (83%), as shown in Table 1.

**Table 1:** Socio-demographic profile of injury patients.

	Unintentional Injuries N = 2955 (100)	RTI N = 1681 (56.8)	Falls N = 692 (23.4)	Burns N = 118 (4.0)	Mechanical N = 195 (6.6%)	Other N = 269 (9.1)	p-value
**Gender**							**<0.001**
Male	2343(79.3)	1359 (80.8)	506 (73.1)	76 (64.4)	167 (85.6)	235 (87.4)	
Female	612 (20.7)	322 (19.2)	186 (26.9)	42 (35.6)	28 (14.4)	34 (12.6)	
**Age (years)**							**<0.001**
0–15	378 (12.8)	122 (7.3)	196 (28.3)	27 (22.9)	22 (11.3)	11 (4.1)	
16–30	1144 (38.7)	711 (42.3)	155 (22.4)	54 (45.8)	100 (51.3)	124 (46.1)	
31–45	735 (24.9)	454 (27.0)	130 (18.8)	24 (20.3)	43 (22.1)	84 (31.2)	
46–60	468 (15.8)	274 (16.3)	125 (18.1)	12 (10.2)	22 (11.3)	35 (13.0)	
>60	230 (7.8)	120 (7.1)	86 (12.4)	1(0.8)	8 (4.1)	15 (5.6)	
**Marital status**							**<0.001**
Married	1106 (37.4)	586 (34.9)	291 (42.1)	51 (43.2)	90 (46.2)	88 (32.7)	
Unmarried	1817 (61.5)	1083 (64.4)	382 (55.2)	67 (56.8)	104 (53.3)	181 (67.3	
Other	32 (1.1)	12 (0.7)	19 (2.7)	0 (0.0)	1 (0.5)	0 (0.0)	
**Education**							**<0.001**
No formal education	747 (25.4)	343 (20.4)	258 (37.6)	37 (31.9)	51 (26.6)	58 (21.6)	
Primary and Middle	912 (31.0)	490 (29.1)	228 (33.2)	46 (39.7)	65 (33.9)	83 (30.8)	
10^th^ year of education and above	1286 (43.6)	848 (50.4)	201 (29.3)	33 (28.4)	76 (39.6)	128 (47.6)	
**Income Quintile**							**<0.001**
Poorest	591 (20.0)	346 (20.6)	137 (19.8)	20 (16.9)	37 (19.0)	51 (19.0)	
Poor	591 (20.0)	290 (17.3)	158 (22.8)	30 (25.4)	61 (31.3)	52 (19.3)	
Middle	591 (20.0)	309 (18.3)	148 (21.4)	33 (28.0)	39 (20.0)	62 (23.0)	
Rich	591 (20.0)	346 (20.6)	123 (17.8)	16 (13.6)	40 (20.5)	66 (24.5)	
Richest	591 (20.0)	390 (23.2)	126 (18.2)	19 (16.1)	18 (9.2)	38 (14.2)	
**Health Insurance status**							**0.05**
Yes	503 (17.0)	268 (16.0)	130 (18.8)	28 (23.7)	39 (20.0)	38 (14.1)	
No	2452 (83.0)	1413 (84.0)	562 (81.2)	90 (76.3)	156 (80.0)	231 (85.9)	
**Type of Hospital (Based on level of care)**							**<0.001**
Secondary care	628 (21.3)	350 (20.8)	174 (25.1)	19 (16.1)	18 (9.2)	67 (24.9)	
Tertiary care	2327 (78.7)	1331 (79.2)	518 (74.9)	99 (83.9)	177 (90.8)	202 (75.1)	
**Hospitalization duration (days)**							**<0.001**
0–3	1398 (47.4)	771 (45.9)	350 (50.6)	38 (32.2)	93 (47.7)	146 (54.3)	
4–6	648 (21.9)	370 (22.0)	145 (21.0)	22 (18.6)	49 (25.1)	62 (23.0)	
7–10	412 (13.9)	250 (14.9)	90 (13.0)	20 (16.9)	24 (12.3)	28 (10.4)	
>10	497 (16.8)	290 (17.3)	170 (15.5)	38 (32.2)	29 (14.9)	33 (12.3)	
Total	2955	1681	692	118	195	269	

*p-*values < 0.05 were considered to be statistically significant

### OOP expenditure and its determinants

**Fig 2:**
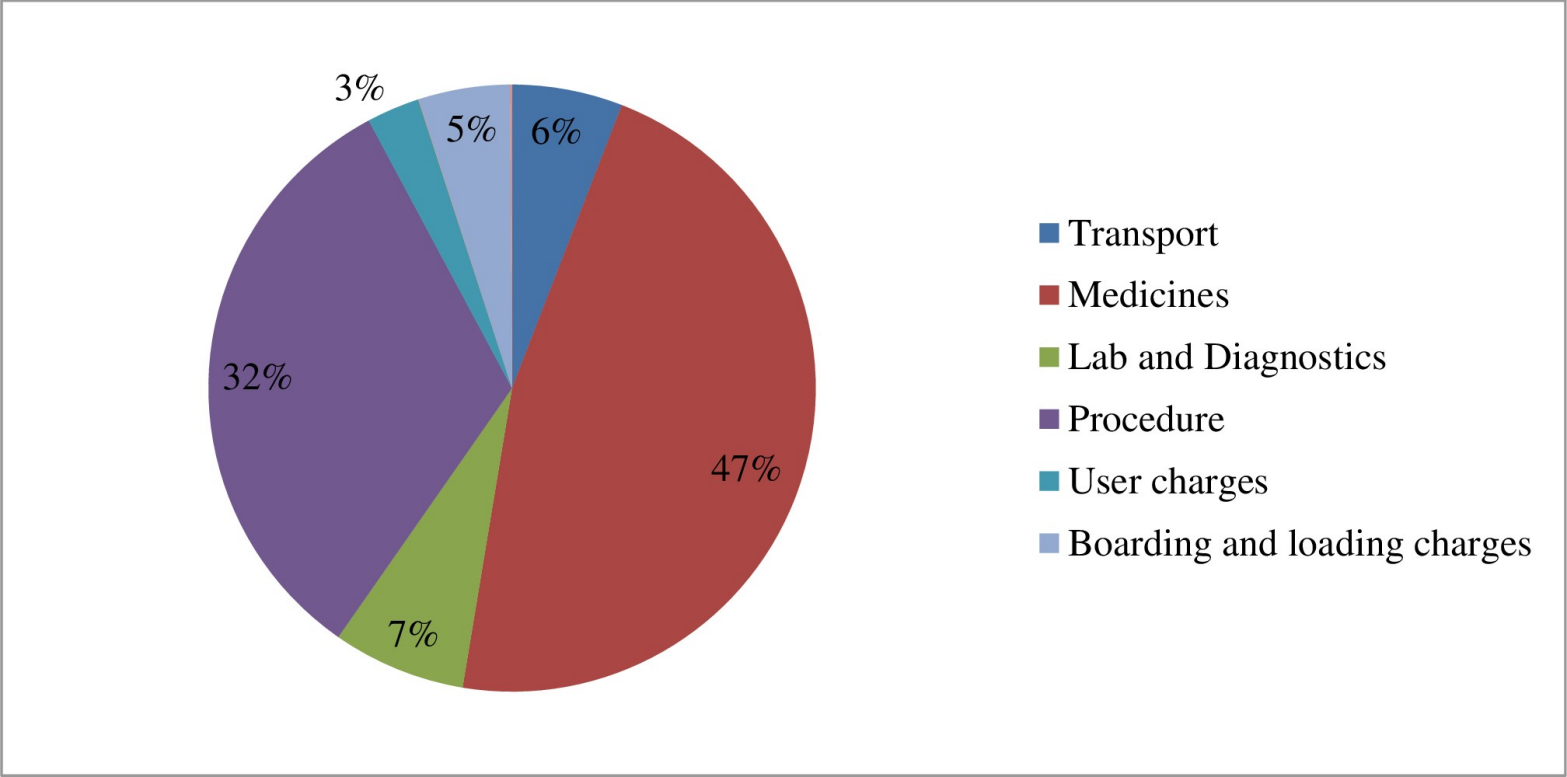
Distribution of out-of-pocket expenditure for injury-related hospitalization.

Mean OOP expenditure per injury-related hospitalization was INR 16,768 (USD 263). Specifically, OOP expenditure was highest for burns, at INR 23,957 (USD 376), followed by RTIs, at INR 17,830 (USD 280), as shown in Table 2. OOP expenditure on medicines was the biggest component, constituting 47% (USD 123) of the total expenses, followed by procedure charges (25%, USD 85.4), (Fig 2). After 12 months following discharge, the overall mean expenditure had increased by 1.5 times (INR 25,420; USD 399) when compared with expenditures during hospitalization. OOP expenditure showed a progressive pattern with the richest quintile incurring higher OOP expenditure (INR 18,060) as compared to the poorest quintile (INR 14,930) as shown in Table 3.

**Table 2:** Out-of-pocket expenditure for injury related hospitalization in three public sector hospitals in North India.

Injury type	Mean (INR,USD)	S.E. Mean (INR, USD)	Median (INR, USD)
RTI	17829.7 (280)	595.4 (9.3)	8000 (125.4)
Falls	15291.2 (240)	884.2 (13.9)	6750 (105.8)
Burns	23957.4 (376)	3374.7 (52.9)	12510 (196.1)
Mechanical injury	14733.7 (231)	1032.1 (16.2)	10020 (157.1)
Other	12257.9 (192)	1034.5 (16.2)	4705 (73.7)
Overall	16768.4 (263)	436.8 (6.8)	7600 (119.1)

**Table 3:** OOP expenditure (INR) for injury-related hospitalization according to socio-economic characteristics of patient.

	Overall	RTI	Falls	Burns	Mechanical	Other	
Variable	Mean (SE)	Mean (SE)	Mean (SE)	Mean (SE)	Mean (SE)	Mean (SE)	p-value
**Gender**							
Male	17085 (485)	18108 (659)	15752 (1012)	25593 (4315)	15670 (1148)	12294 (1117)	0.156
Female	15556 (1003)	16655(1387)	14038 (1801)	20997 (5416)	9149 (1903)	12009 (2767)	
**Age (years)**							
0–15	10505 (1089)	15686 (2817)	6775 (972)	12157 (2617)	11632 (2024)	13202 (4982)	**<0.001**
16–30	17840 (741)	17893 (894)	19316 (2200)	30592 (6429)	14804 (1493)	12582 (1727)	
31–45	17790 (870)	18360 (1178)	19682 (2063)	20194 (5277)	18138 (2455)	10914 (1725)	
46–60	18806 (1130)	18311 (1409)	21910 (2604)	29926 (9301)	10197 (2503)	13198 (2181)	
>60	14323 (1158)	16528 (1791)	11189 (1638)	3020 (0)	16563 (4910)	14217 (3266)	
**Marital status**							
Married	15399 (687)	17071(1002)	11662 (1289)	20076 (3289)	14205 (1559)	15141 (2273)	**0.003**
Unmarried	17416 (556)	18227 (744)	17132 (1085)	26912 (5386)	15310 (1387)	10856 (1059)	
**Education**							
No formal education	12258 (1035)	15052 (1121)	10388 (1038)	14500 (2430)	13176 (2084)	9383 (1655)	**<0.001**
Primary and Middle	12846 (671)	18316 (1197)	14941 (1489)	19048 (3621)	16026 (1626)	11945 (1804)	
10^th^ year of education and above	16766 (793)	18672 (840)	21968 (2060)	42529 (10006)	14875 (1770)	13764 (1667)	
**Quintile**							
Poorest	14930 (874)	15399 (1092)	15511 (2210)	20923 (5536)	10404 (1707)	11124 (2442)	0.105
Poor	15982 (899)	17018 (1417)	16551 (1707)	18324 (4140)	15263 (2049)	7970 (1634)	
Middle	18043 (1004)	20264 (1617)	14159 (1640)	19449 (2957)	16618 (2285)	16393 (2469)	
Rich	16827 (900)	18950 (1182)	12608 (2157)	24598 (7466)	15782 (2526)	12308 (1964)	
Richest	18060 (1172)	17667 (1334)	17422 (2260)	43337(17023)	15429 (2833)	12814 (3062)	
**Health Insurance (status)**							
Yes	16346 (482)	17476 (646)	14800 (1004)	23239 (4082)	14412 (1187)	14913 (3151)	**0.033**
No	18827 (1031)	19697 (1524)	17416 (1820)	26265 (5582)	16019 (2038)	14734 (1032)	
**Type of Hospital (Based on level of care)**							
Secondary	6390 (447)	6526 (413)	7072 (1335)	2773 (655)	6770 (1986)	6390 (477)	**<0.001**
Tertiary	19569 (527)	20802 (722)	18052 (1066)	28023 (3891)	15544 (1102)	19569 (437)	
**Hospitalization duration (days)**							
1	3882 (226)	3857 (333)	3512 (381)	3919 (1456)	7459 (1173)	3197 (448)	**<0.001**
2	5542 (361)	5510 (487)	5153(680)	6963 (1423)	6101 (968)	5607 (1499)	
3	10946 (757)	11347 (1036)	10986 (1664)	5517 (3085)	10923 (1704)	9659 (2584)	
4	13184 (925)	12764 (1107)	11897 (2078)	6020 (1766)	15064 (1942)	18970 (5357)	
5	16768(1218)	18481 (1702)	17343 (3031)	8491 (1405)	17172 (3645)	19448 (4896)	
>5	31985 (1027)	33154 (1316)	30647 (2181)	45360 (8078)	23737 (2446)	31985 (1027)	

*p-*values < 0.05 were considered to be statistically significant

### Indirect costs of hospitalization due to injuries

Mean indirect cost due to injury-related hospitalization according to HCA and FCA was estimated to be INR 8,164 and INR 2,870 respectively (Table 4). Annual indirect cost per injured case according to HCA and FCA was found to be INR 28,257 and INR 7,178 respectively. The annual indirect costs are as much as the annual direct costs of injuries (INR 25,420), thus showing the extent of the total cost of an injury. Further, after accounting for productivity loss of the patients who died as a result of injury, the indirect costs per injured case increased to INR 3,028,435 and INR 35,237 according to HCA and FCA respectively. Additionally, we also calculated indirect costs using HCA without assuming a minimum wage rate for those not a part of the workforce. In this case the mean indirect costs for injury-related hospitalization and annual indirect costs per injured case were estimated to be INR 2,870 and INR 9,889 respectively. Further, after accounting for patients who died as a result of injury, the indirect costs per injured case were limited to INR 1,433,323 instead of INR 3,028,435 as estimated previously.

**Table 4:** Mean OOP expenditure and mean Indirect costs due to injury-related hospitalizations.

Timeline	Mean OOP expenditure	Mean Indirect Costs	
		Human Capital Approach	Friction Cost Approach*
Hospitalization	16768	8164	2870
Follow-up 1	5373	10341	2576
Follow-up 2	1813	7649	1426
Follow-up 3	936	3364	967
Follow-up 4	430	5582	0

*The estimates for FCA are at the monthly level taking a friction period of 3 months

### Catastrophic Health Expenditure (CHE)

The prevalence of catastrophic health expenditure for hospitalization due to injuries at 30% cut-off was 22.2%. As expected, the prevalence of CHE increased to 30.7% at a lower threshold of 20% and decreased to 16.2% at a higher threshold of 40%. Poverty head count as a result of injury-related hospitalization was 12.2%. Prevalence of CHE and impoverishment were significantly higher for poorest quintile, seeking care at tertiary care hospital and increased duration of hospitalization (p< 0.001) as shown in Table 5. Table 6 reports odds of facing CHE and impoverishment estimated using logistic regressions. It was seen that wealth quintiles, type of hospital and duration of hospitalization are the significant determinants of CHE and impoverishment.

**Table 5:** Prevalence of catastrophic health expenditure and impoverishment among injury-related hospitalization.

Category	Prevalence of Catastrophic Health Expenditure (CHE)	p value	Prevalence of Impoverishment	p-value
***Gender***		**0.01**		0.06
Male	22.7		13.1	
Female	20.2		9.1	
***Age groups***		**<0.001**		**0.01**
0–15	12.3		8.8	
16–30	22.9		13.0	
31–45	25.5		14.5	
45–60	26.0		16.4	
Above 60	16.7		4.4	
***Marital status***		**0.04**		0.09
Unmarried	19.9		9.5	
Married	23.4		13.4	
Other	31.3		21.4	
***Education***		**0.004**		0.07
No formal education	18.4		9.0	
Primary and Middle	21.8		13.3	
10^th^ year of education and above	24.8		13.9	
***Wealth quintiles***		**<0.001**		**0.004**
Poorest	28.4		14.0	
Poor	24.2		12.4	
Moderate	25.9		13.4	
Rich	19.2		11.5	
Richest	13.4		5.3	
**Health Insurance**		0.012		0.03
Yes	26.1		16.4	
No	21.4		9.0	
**Hospitalization duration (days)**		**<0.001**		**<0.001**
0–3	5.1		**3.9**	
4–6	19.4		11.9	
7–10	36.7		17.9	
>10	61.9		33.6	
**Type of hospital**		**<0.001**		**<0.001**
Secondary level	3.8		3.6	
Tertiary level	27.3		15.1	
**Type of injury**		**0.006**		0.06
RTI	23.5		13.9	
Falls	19.1		9.9	
Burns	31.6		19.3	
Mechanical Injury	22.5		11.4	
Other	17.7		6.9	
Overall	22.2		12.2	

*p-*values < 0.05 were considered to be statistically significant

**Table 6:** Determinants of catastrophic health expenditure and impoverishment for injury-related hospitalization.

Category	CHE Adjusted O.R. (95% C.I.)	p-value	Impoverishment Adjusted O.R. (95% C.I.)	p-value
***Age groups***				
0–15	1	**0.002**	1	0.16
16–30	1.8 (1.2–2.8)		1.1 (0.5–1.7)	
31–45	1.9 (0.8–2.2)		1.1 (0.6–1.8)	
45–60	2.5 (1.5–4.6)		1.6 (0.8–3.3)	
Above 60	2.7 (1.4–4.5)		0.4 (0.1–1.3)	
***Gender***				
Female	1	0.79	1	0.33
Male	1.1 (0.7–1.5)		1.3 (0.8–2.1)	
***Education***				
No formal education	1	0.14	1	0.25
Primary and Middle	0.9 (0.6–1.3)		1.3 (0.8–2.1)	
10^th^ year of education and above	0.7 (0.5–1.1)		1.6 (0.9–2.8)	
***Wealth quintiles***				
Poorest	1	**<0.001**	**1**	**0.01**
Poor	0.65 (0.4–0.9)		1.3 (0.7–2.2)	
Moderate	0.63 (0.4–0.9)		0.8 (0.4–1.4)	
Rich	0.56 (0.4–0.8)		0.9 (0.5–1.5)	
Richest	0.38 (0.2–0.6)		0.3 (0.2–0.7)	
**Health Insurance**		0.53		0.69
Yes	1.2 (0.8–2.4)		1.4 (0.7–2.5)	
No	1		1	
**Type of Hospital**		**<0.001**		**<0.001**
Secondary	0.4(0.3–0.6)		0.3 (0.1–0.5)	
Tertiary	1		1	
**Hospitalization duration**		**<0.001**		**<0.001**
0–3	1		1	
4–6	1.7 (1.1.-2.4)		3.1 (1.7–5.6)	
7–10	2.0 (1.3–3.0)		5.1 (2.8–9.1)	
>10	5.3 (3.5–7.9)		12.2 (7.1–20.9)	
**Type of injury**		0.73		0.48
RTI	1		1	
Falls	0.91 (0.7–1.8)		0.9 (0.6–1.5)	
Burns	0.93 (0.5–1.8)		1.3 (0.6–2.9)	
Mechanical Injury	1.1 (0.6–2.0)		0.7 (0.3–1.7)	
Other	0.79 (0.6–1.4)		0.5 (0.2–1.2)	

Number of observation: 2955

*p-*values < 0.05 were considered to be statistically significant

## Discussion

Injuries are a major public health problem in India [30]. The present study was conducted to identify economic impact of injuries in terms of out-of-pocket expenditure and prevalence of catastrophic health expenditure for patients hospitalized due to different forms of injuries. Overall it was estimated that direct OOP expenditure for hospitalizations was INR 16,768 (USD 263) while the indirect productivity loss was INR 8,164 (USD 128). The prevalence of catastrophic expenditure was 22.2% with about 12.2% slipping below poverty line as a result of costs incurred.

### Findings in context of existing evidence

Our estimate of mean OOP expenditure per hospitalization due to injury is less than that reported in the recently conducted National Sample Survey 71^st^ round on health care service utilization and expenditures (INR 23,491 per hospitalization case) [7]. This is likely due to the fact that we recruited all our patients from secondary and tertiary level public sector hospitals, while NSSO data comprise mix of public as well as private sector hospitalizations due to injuries. On an average OOP expenditures in the private sector are almost five times higher than those in the public sector (INR 36,255 and INR 6,729 respectively) [7]. Further it was found that mean OOP expenditure after 12 months following discharge had increased by 1.5 times when compared with expenditures during hospitalization. This is in line with our earlier study which reports a slightly higher increase in mean OOP after 12 months (2.5 times) as it was undertaken at tertiary care level only [11].

OOP expenditure per hospitalization case was found out to be highest for burns (USD 376) followed by RTI (USD 280), which is coherent with other studies showing that burns (USD 427) account for maximum OOP expenditure out of the all forms of injuries [31]. The same study reported OOP expenditure for RTI as USD 367 while another study conducted specifically for RTI reported OOP as USD 363 [32].

Medicines accounted for nearly half of the total OOP payments (47%) made by the patients hospitalized due to injuries. This is corroborated by numerous studies stating medicines form a large component of total OOP expenditure [33–35]. To curtail such expenses the public health system needs to be strengthened by ensuring a good supply chain to meet demands for drugs and diagnostics [36].

The prevalence of catastrophic health expenditure was 22.2%. Prevalence of CHE was significantly more with increase in age, poorest quintile, hospitalization in tertiary care hospital and increased duration of hospitalization (p< 0.001). The odds of facing CHE were highest among elderly (<60 years) as they incur higher OOP expenditures owing to pre-existing functional disabilities and physical conditions [37]. Also the prevalence of CHE and impoverishment increased with increasing duration of hospitalization. These findings are similar to a study conducted in Vietnam which also reported longer hospital stay, older age, lower income to be significantly associated with increased risk of CHE [31]. Further, the odds of facing CHE and impoverishment were higher for those hospitalized at tertiary care level as compared to those at secondary care level because higher OOP expenditures incurred at the former. The difference in OOP expenditure may be attributed to the difference in the case mix of the patients (more severe patients receive care at tertiary level institutions), the intensity of resource use, the skill mix of healthcare professionals (highly skilled at tertiary care level).

It was seen that while OOP expenditure showed a progressive pattern (higher OOP as a proportion of income for the richest quintile), the effect of impoverishment was regressive (significantly higher for the poorest quintile). Similar results have been reported by previous studies conducted for injuries specifically or CHE due to hospitalization in general [8], [38]. These results highlight a great degree of inequity in the distribution of impoverishing effects of injury-related hospitalization across income quintiles. A number of reasons have been cited in the literature to explain these findings. Firstly, a significantly large proportion of the Indian population lies just above the poverty line. For them, even lower amount of OOP expenditure results in the household being pushed below poverty line. Secondly, lack of efficient risk pooling mechanisms results in higher impoverishment rates. Reducing such inequalities is one of the fundamental objectives under universal health coverage. Further, it was seen that being covered under a health insurance scheme did not guarantee protection from facing CHE. These findings related to effectiveness of health insurance for reducing OOP expenditure and CHE are similar to what has been reported in a recently published systematic review of impact evaluation of large publicly financed health insurance schemes [39]. This review reports that the majority of insurance schemes, while increasing the utilization of healthcare services, do not result in a significant reduction in OOP expenditure or improvement in prevalence of CHE. A number of reasons have been cited to explain this paradoxical finding. Firstly, it could be due to the significantly lower benefit package which results in people spending out of pocket from their side. Secondly, due to poor regulation private hospitals continue the practice of balance billing to the patients. Thirdly, lack of awareness among insured population results in patients accessing care from providers who are not covered under the insurance scheme. In the case of our study the public sector hospitals are not covered under any health insurance scheme. To overcome these issues, the recently launched *Pradhan Mantri Jan Aarogya Yojna (PMJAY)*, has incorporated a much higher benefit package (INR 500,000) and proposes a management information system to track practices of private practitioners as well as patient satisfaction [40]. Further, various cashless insurance schemes designed specifically for road accident victims have been started by the central and state government [41–43]. An early evaluation of one such scheme, the ‘Cashless scheme of health insurance for road accident victims’ implemented on pilot basis by the Ministry of Road Transport and Highways (MoRTH), Government of India (GoI) shows a reduction in OOP expenditure and FRP among those who were a beneficiary of the scheme in comparison to those who were not entitled to receive the benefits of this scheme [41]. These findings imply the need for targeted risk pooling mechanisms and free delivery of services at the point of contact.

The mean indirect cost of injury-related hospitalization according to HCA and FCA was INR 1,853 and INR 28,704 respectively. According to neoclassical economic theory, the concept of productivity is based on the production function, where output is a function of capital input, labor input and technology allowing for substitution between different types of inputs. Productivity is a measure of output per unit of input [38]. In this context productivity loss is the output lost corresponding to lost input due to injury-related hospitalization. It is the value of time for those activities which are foregone by the patient and his/her caregiver while the patient is hospitalized. In this case input is measured in terms of income per working day foregone [26].We found that while the OOP expenditure was higher during the hospitalization period as compared to follow-ups, the reverse was true for indirect costs. Majority of the OOP expenses are incurred during the hospitalization period as major surgical procedures, diagnostic tests etc occur at that time and relatively lesser expenditure related to drugs and follow-up visits were incurred as part of post-discharge OOP spending at different follow-up intervals. On the other hand, indirect costs were higher when calculated at different follow-ups as compared to hospitalization period because more working days were lost during the former. While we obtained a higher value of indirect costs when calculated by HCA, it might be seen as an overestimation. Contrary to the HCA, which takes a societal perspective, FCA calculations are based on employers’ perspective. In FCA it is assumed that the work of those who are absent due to illness/ death is taken up by those who are unemployed within a fixed period of time referred to as the friction period [28]. We provide results based on both the approaches, thus providing a comprehensive picture of indirect costs.

### Strengths

First, we report economic burden for all injuries and we are not confined to any one particular injury type (e.g. RTI). Second, the data collection for injury-related expenditure covered both the period of hospitalization as well as multiple follow ups during the post-discharge period (up to 12 months post injury). Third, apart from measuring the direct OOP payments made by the patient, we also collected data from patients and their caregivers to estimate indirect costs due to injuries. Thus, the overall sum of direct OOP expenditure and indirect costs give a comprehensive picture of economic burden due to injuries.

Most of the previous studies assessing the OOP expenditures for injury have relied on community based identification of trauma cases who sought ambulatory care in last 15 days or hospitalization in last 1 year [7], [44]. Such interviews are usually limited in terms of recall bias. Secondly, these are usually cross-sectional and do not collect information on long-term costs. Our methodology of data collection improves these weaknesses of previous studies. In our study, cost-related data to estimate OOP expenditure were collected on a day-to-day basis during hospitalization with a recall period of only 24 hours. Another strength of our study lies with high follow up rates. Follow-up interview completion rates at each wave were over 80%, adding to the robustness of our findings.

### Limitations

We acknowledge certain methodological limitations in our study. First, our study is based on self-reporting of treatment and expenditure related data. However, wherever possible our investigators tried to corroborate the findings with hospital and other related bills. Further, we could not elicit data on consumption expenditure in about 28% of the patients. Second, our sample was facility-based, recruited from public sector hospitals only, which may not give an overall comprehensive picture. However, we recruited patients both from secondary level l and tertiary level hospitals. Besides receiving new cases, the tertiary hospital also serves as a referral centre for other hospitals in the neighboring four states. Further, since we included only hospitalized cases, a limitation of our study is the inability to estimate the cost of ambulatory care. However, among the hospitalized patients we also collected OOP expenditure after discharge which included ambulatory care. Further, we did not assess the severity of illness since standard methods of assessing clinical severity, such as injury severity score (ISS), are not routinely recorded in clinical case sheets maintained by health care personnel in India. As a result of this limitation clinical assessment of severity was not part of data collection. However, we have presented results with respect to length of stay, which is an indirect measure for the severity of injury. Lastly, we did not collect data on direct non-healthcare costs including those associated with vehicle and other property damage and costs associated with police attendance and investigation of crashes, or other legal fees. This may have resulted in an underestimation of the overall economic loss due to injuries as reported by us.

### Conclusion

The economic impact of injuries is significantly high in India both in terms of OOP expenditure and productivity losses. Availability of drugs and diagnostics needs to be strengthened in the public health sector to ensure equitable access to treatment. These results are important and should inform health care financing reforms in India as well as other countries having similar settings.

## Supporting information

S1 Dataset Anonymized study dataset. (SAV)
